# EHMTI-0273. Onabotulinotoxin type A is a therapeutic option in chronic cluster headache

**DOI:** 10.1186/1129-2377-15-S1-C51

**Published:** 2014-09-18

**Authors:** N Mas-Sala, M Quintana, J Alvarez-Sabin, P Pozo-Rosich

**Affiliations:** 1 Neurology, Hospital Universitari Vall d'Hebron, Barcelona, Spain

## 

To analyse the efficacy of Onabotulinumtoxin type A (OnabotA) in chronic cluster headache patients.

Six patients (5 men and 1 woman) diagnosed with refractory chronic cluster headache were included. Data regarding the characteristics of the headache, the frequency and the duration of the attacks, the presence of family history of migraine and the analgesic use were collected. The patients continued with their usual preventative treatment.

In 4 of the 6 patients included (66.6%), we have observed a clear improvement in the frequency of the headache with a change in the pattern (from chronic to episodic). All of these had family history of migraine. In one of the patients no improvement was observed, and one patient was lost during follow-up.

The use of OnabotA could be considered as a therapeutic option in those patients with chronic cluster headache which are intolerant or refractory to the known and used preventative treatments, before using more invasive treatments. Larger series are needed to confirm these findings.

